# Advances in transcorneal electrical stimulation: From the eye to the brain

**DOI:** 10.3389/fncel.2023.1134857

**Published:** 2023-03-02

**Authors:** Stephen K. Agadagba, Lee Wei Lim, Leanne Lai Hang Chan

**Affiliations:** ^1^Centre for Eye and Vision Research Ltd., Hong Kong, Hong Kong SAR, China; ^2^Neuromodulation Laboratory, School of Biomedical Sciences, Li Ka Shing Faculty of Medicine, The University of Hong Kong, Pokfulam, Hong Kong SAR, China; ^3^Department of Electrical Engineering, City University of Hong Kong, Kowloon Tong, Hong Kong SAR, China

**Keywords:** neuromodulation, transcorneal electrical stimulation, eye-brain connection, brain neural oscillations, electrophysiology, plasticity

## Abstract

The mammalian brain is reported to contain about 10^6^–10^9^ neurons linked together to form complex networks. Physiologically, the neuronal networks interact in a rhythmic oscillatory pattern to coordinate the brain’s functions. Neuromodulation covers a broad range of techniques that can alter neuronal network activity through the targeted delivery of electrical or chemical stimuli. Neuromodulation can be used to potentially treat medical conditions and can serve as a research tool for studying neural functions. Typically, the main method of neuromodulation is to electrically stimulate specific structures in both the central and peripheral nervous systems *via* surgically implanted electrodes. Therefore, it is imperative to explore novel and safer methods for altering neuronal network activity. Transcorneal electrical stimulation (TES) has rapidly emerged as a non-invasive neuromodulatory technique that can exert beneficial effects on the brain through the eyes. There is substantial evidence to show that TES can change the brain oscillations in rodents. Moreover, the molecular data clearly shows that TES can also activate non-visual brain regions. In this review, we first summarize the use of TES in the retina and then discuss its effects in the brain through the eye-brain connection. We then comprehensively review the substantial evidence from electrophysiological, behavioral, and molecular studies on the role of TES on modulating neurons in the brain. Lastly, we discuss the implications and possible future directions of the research on TES as a non-invasive tool for neuromodulation of the brain *via* directly stimulating the mammalian eye.

## 1 Introduction

The brain is one of the most complex and important organ in the body responsible for maintaining homeostasis and coordinating bodily functions. The mammalian brain is composed of about 10^6^–10^9^ neurons in a complex hierarchical network (Herculano-Houzel, 2009; Herculano-Houzel et al., 2011). These neurons communicate with each other in many ways, one of which is through neural oscillations. Neural oscillations are repetitive or rhythmic patterns of neuronal activity generated throughout the central nervous system (CNS). These neural oscillations operate at varying frequencies and can be classified into six frequency bands: slow oscillations (0.1–4 Hz), delta (1–4 Hz), theta (4–7 Hz), alpha (7– 13 Hz), beta (14–30 Hz), and gamma (>30 Hz). The synchronized oscillations originate either from the activity of single neurons or from the interactions in the neuronal network. At the single neuron level, neural oscillations reflect the fluctuations in the resting potential or the rhythmic action potentials, which then trigger neural oscillations in post-synaptic neurons. On the other hand, at the neuronal network level, the synchronized activity of neural ensembles evokes neural oscillations at various frequency bands at the macroscopic scale, which can be measured by techniques such as electroencephalography (EEG), electrocorticography (ECoG), and magnetoencephalography (MEG). Neural oscillations in the brain have been widely studied in various areas of scientific research, particularly in cellular and cognitive neuroscience. Growing evidence suggests that neural oscillations are pivotal for neural communication. The synchronous activity of neurons generates rhythms that enable different brain regions to communicate and coordinate responses to both intrinsic and extrinsic stimuli. For instance, two groups of neurons in phase lock or phase synchrony can influence each other because they are simultaneously in an excited state and have strong communication with each other. However, neurons that are desynchronized have weak communication and do not effectively orchestrate responses to stimuli. Indeed, disruption of brain rhythms and neuronal synchrony have been implicated in the pathophysiology of various neurodegenerative disorders and neurological diseases including Parkinson’s disease (PD), Alzheimer’s disease (AD), and epilepsy (Buzsáki, 2015). In patients with PD, the progressive death of dopaminergic neurons in the substantia nigra causes circuitopathy, which leads to hypersynchronization of beta oscillations in the substantia nigra and other brain structures such as the globus pallidus interna, globus pallidus externa, and subthalamic nucleus (Chan et al., 2021). Similarly, network abnormalities including hypersynchrony and altered oscillatory activity in AD (Buzsáki, 2006; Palop and Mucke, 2016) and increased neuronal hyperexcitability in epilepsy (Adaikkan et al., 2019) have also been reported. Exploring these dysfunctional brain networks raises the possibility of using neuromodulation to normalize the oscillatory relationships and restore healthy neuronal communication.

Neuromodulation is a neurostimulation technique that alters neuronal network activity by the targeted delivery of stimuli in specific regions of the body. The underlying aim of neuromodulation is to modify abnormal circuitry to restore the normal physiological state (Herrington et al., 2016). Neuromodulation has been used in a wide range of applications including altering the activity of dysfunctional neuronal networks, as a potential medical therapy, and as a research tool for studying neural functions. Most neuromodulatory stimulation methods are electricity-based, although alternative stimulation methods also include the use of magnetic fields, optogenetics, and focused ultrasound. Generally speaking, neuromodulatory stimulation can be divided into either invasive and non-invasive methods. The major difference between invasive and non-invasive neuromodulation is that the former involves the introduction of a stimulation tool or electrode into the body cavity, whereas the latter is performed outside the body. Some examples of invasive neuromodulation include deep brain stimulation (DBS), spinal cord stimulation, and stimulation methods that target specific nerves such as the vagus or sacral nerves. These invasive approaches have been reported to have several beneficial effects. For example, clinical results from four randomized control trials conducted to determine the effects of DBS in Parkinson’s disease showed that it significantly reduced the need for oral levodopa treatment while limiting dyskinesias by improving levodopa “On” time and reducing the “Off” time (Deuschl et al., 2006; Smeding et al., 2006; Weaver et al., 2009; Williams et al., 2010). The use of invasive neuromodulation as potential treatment options for chronic pain, urinary incontinence, epilepsy, and depression have also been reported. On the other hand, early non-invasive neuromodulatory approaches were unspecific. For example, electroconvulsive therapy developed in the 1930 s for treating patients with schizophrenia delivered the electrical stimulation to large areas of the brain. Subsequently, non-invasive neuromodulatory approaches have become more refined and novel approaches have been developed that can stimulate spatially distant regions of the brain. Some examples include transcranial direct current stimulation (tDCS), transcranial alternative current stimulation (tACS), and transcranial magnetic stimulation (TMS). The US Food and Drug Administration (FDA) has approved the clinical use of TMS in humans for alleviating symptoms in several mental health conditions, whereas the use of tDCS and tACS are still under investigation and awaiting approval.

There are inherent differences in the neurosurgical and stimulation risks between invasive and non-invasive neuromodulation. Depending on the specific invasive neuromodulatory technique, post-surgical complications and invasive stimulation-related adverse effects can occur, including neurological sequelae such as infection, intracranial hemorrhage, post-procedural seizures, and cognitive impairment, particularly decline in verbal fluency (John et al., 2021; Xu et al., 2021), and psychiatric side effects such as apathy, frontal disinhibition, and suicide in rare cases (Smeding et al., 2006). Although non-invasive neuromodulation does not require surgery and the stimulation is relatively painless with minimal side effects, there have been reported cases of minor adverse effects from non-invasive methods such as TMS and tDCS. These minor adverse effects, including headaches, scalp discomfort, fatigue, redness at the stimulated site, tingling, and itching sensations, were reported to be naturally resolved without the need for any medical intervention (Krishnan et al., 2015; Matsumoto and Ugawa, 2017). Nevertheless, exploring safe novel methods for altering neuronal network activity in the brain is imperative for treating diseases and improving overall brain health. Ideally, such non-invasive methods should have minimal side effects, while achieving its therapeutic goal or serving as a tool for investigative purposes. Transcorneal electrical stimulation (TES) is rapidly emerging as a safe non-invasive neuromodulatory technique that can exert beneficial effects in the brain *via* stimulating the eyes.

In this review, we discuss the advances in neuromodulation by TES in relation to the eye-brain connection. We summarize the conventional application of TES in the retina as a treatment for ocular disorders. We then give an in-depth explanation of the eye-brain connection and the possible mechanism of the neuromodulatory effects of TES in the brain. Lastly, we present the implications and possible future directions of TES as a tool for non-invasive brain neuromodulation. In the subsequent sections of this review, we use the term neuromodulation in the context of the alteration of neuronal network activity by TES, unless otherwise stated.

## 2 Conventional applications of TES of the retina

**Figure 1:**
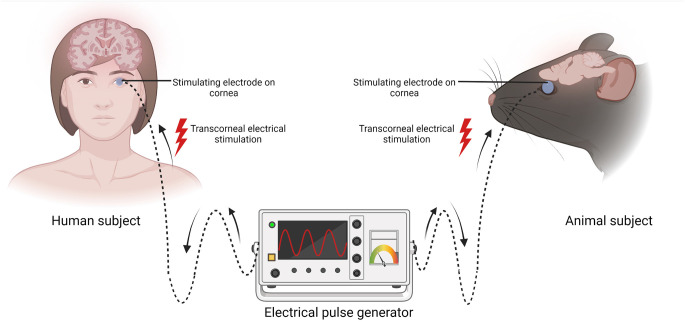
Schematic representation of transcorneal electrical stimulation (TES). In both human and animal subjects, the stimulating electrode (blue circle) is positioned on the cornea and the other end is connected to an electrical pulse generator (EPG). Electric current pulses activate the retina and its downstream structures. Created with BioRender.com (accessed December 13, 2022).

Transcorneal electrical stimulation is a non-invasive method of activating the retina and retinofugal pathways. In practice, an active electrode is placed on the corneal surface of the eye and an inactive reference electrode is carefully positioned underneath the skin in close proximity to the eye. The active electrode is connected to an electrical pulse generator that controls the delivery of electric current to the eye (Figure 1). The stimulation parameters such as current amplitude, stimulation frequency, pulse duration, and repetition periods can be adjusted and varied based on each subject and pathological condition. Several studies have shown that TES has immense neuroprotective benefits in retinal trauma and degeneration and in healthy subjects, as demonstrated in the animal studies in Table 1 (Morimoto et al., 2005, 2010; Ni et al., 2009; Tagami et al., 2009; Wang et al., 2011; Henrich-Noack et al., 2013b; Fu et al., 2018; Jassim et al., 2021) and the clinical studies in Table 2 (Inomata et al., 2007; Kurimoto et al., 2010; Oono et al., 2011; Schatz et al., 2011, 2017; Naycheva et al., 2013). These studies have provided crucial evidence for the therapeutic effectiveness of TES in the retina and have also examined the potential underlying mechanisms. Below is a summary of the conventional neuroprotective roles of TES in the retina.

**Table 1:** Summary of preclinical studies on the effects of TES of the retina.

**Study**	**Experimental subject; sample size**	**Control groups; sample size**	**Health state**	**TES parameters**	**TES duration**	**Effects**
Morimoto et al. (2005)	Wistar rats; 26	Wister rats (sham); 12	Transected optic nerve	100 μA, 20 Hz, and 3 ms/phase	Single session for 60 min	TES facilitated survival of axotomized RGCs based on the strength of the electric charge. Increased IGF-1 expression in the retina.
Ni et al. (2009)	SD rats; 332	SD rats (light stimulation)	Degenerated photoreceptors	100–500 μA, 20–100 Hz, and 3 ms200 or 300 μA, 20 Hz, and 3 ms	Single session for 90 min Single session/3 days for 14 days	Neuroprotective effects after TES stimulation. TES induced upregulation of Bcl-2, CNTF, and BDNF. TES induced downregulation of Bax.
Tagami et al. (2009)	Wistar rats; 36	Wistar rats (sham); 12	Crushed optic nerve	100 μA, 20 Hz, and 1 ms/phase	60 min of varied TES applications: day 0 (1× TES), day 0 and 7 (2× TES), day 0, 4, 7 and 10 (4× TES), days 0–12 (TES daily)	TES induced RGC survival and axonal regeneration following optic nerve crush.
Morimoto et al. (2010)	Wistar rats; 36	Wistar rats (sham); 12	Transected optic nerve	100 μA; 20 Hz; 0.5, 1, 2, 3, and 5 ms/phase 50, 100, 200, 300, and 500 μA; 20 Hz; 1 ms/phase 100 μA; 0.5, 1, 5, 20, 50; and 100 Hz; 1 ms/phase	60 min each time. Repeated stimulation data (on days 0, 4, 7 and 10) were compared with single stimulation data (14 days post-transection)	Optimal parameters for retinal neuroprotection were 1 and 2 ms/phase; 100 and 200 μA; 1, 5, and 20 Hz. Greater than 30 min stimulation was needed to attain neuroprotective effects. Repetitive TES was more effective than single TES post-transection.
Wang et al. (2011)	SD rats; 28	SD rats (sham)	Optic nerve ischemia	300 μA, 20 Hz, and 3 ms/phase	60 min/2 days for 14 days	Day 7 and day 14 post-ischemia showed higher RGC density in TES-treated rats. TES preserved retinal thickness.
Henrich-Noack et al. (2013b)	Hooded rats; 8	Hooded rats (sham); 8	Crushed optic nerve	100 μA, 20 Hz, and 1 ms/phase	60 min immediately after optic nerve crush (ONC) 60 min on day 11 post-ONC	Increased RGC survival in TES-treated rats. Morphological changes in preserved neurons were milder in TES-treated rats.
Fu et al. (2018)	Mongolia gerbils; 15	Mongolia Gerbils (sham); 15	Ocular ischemia leads to elevated intraocular pressure (IOP)	100 μA, 20 Hz, and 1 ms/phase	60 min/session First session: immediately post-IOP elevation Second session: 4 days post-IOP Afterwards, two sessions/week for 1 month	TES enhanced scotopic b-wave and photopic negative response amplitude, signifying increased bipolar and RGC survival after ischemia. Decreased microglial cell number and increased IL-10 expression, signifying anti-inflammatory response in the retina of TES-treated gerbils.
Jassim et al. (2021)	DBA/2J mice; 18	DBA/2J mice (sham); 25	Secondary glaucoma	100 μA, 20 Hz, and 1 ms/phase	10 min/session One session/3 days for 2 months	TES induced axon survival but had no effect on RGC survival and IOP. TES induced decrease in Iba-1a + microglia expression in the retina, signifying anti-inflammatory action.

TES, transcorneal electrical stimulation; RGCs, retina ganglion cells; IGF-1, insulin-like growth factor 1; CNTF, ciliary neurotrophic factor; BDNF, brain-derived neurotrophic factor; IL-10, interleukin-10; Iba-1a, ionized calcium-binding adapter molecule 1a; Bax, bcl-2-associated × protein, 1×, 2×, 3×, and 4× represent one, two, three and four applications respectively.

**Table 2:** Summary of clinical studies on TES effects in the retina.

**Study**	**Experimental subject; sample size**	**Control groups; sample size**	**Health state**	**TES parameters**	**TES duration**	**Effects**
Inomata et al. (2007)	Humans; 3	Humans (fellow eye); 3	RAO	0–1,000 μA until phosphenes were reported and 20 Hz	30 min per session Once monthly for 3 months duration	TES induced improvement in retinal function. All subjects demonstrated improved visual field. Two out of three subjects showed improved visual acuity and multifocal ERG.
Oono et al. (2011)	Humans; 5	N/R	Branch RAO	500–900 μA and 20 Hz	30 min per session	TES promoted reformation of the visual function specifically in long-standing branch RAO cases.
Schatz et al. (2011)	Humans; 24	Humans (sham); 24	RP	66% and 150% of individual subject’s electrical phosphene threshold (EPT), 20 Hz, and 5 ms/phase	6 weeks (30 min/week)	TES improved visual field. Visual functions in the 150% group either remained unchanged or improved after TES. TES decrease visual field mean sensitivity and desaturated color discrimination. 66% of group displayed no marked tendency following TES.
Naycheva et al. (2013)	Humans; 13	Humans (sham); 3	Central and branch RAO	Biphasic pulses (5 ms cathodic then 5 ms anodic), 66% and 150% of individual subject’s EPT, and 20 Hz	6 days (30 min/day)	150% group showed increase in scotopic a-wave slopes after TES. ERG parameters remained unchanged in the other group after TES.
Kurimoto et al. (2010)	Humans; 10	Humans (fellow eye; sham) 10	Healthy normal-sighted	150 μA and 20 Hz	30 min per session	Enhanced chorioretinal blood flow in normal-sighted subjects after TES. TES caused only mild effects on IOP and systemic blood circulation.

TES, transcorneal electrical stimulation; ERG, electroretinogram; RAO, retinal artery occlusion; RP, retinitis pigmentosa; N/R, not reported.

### 2.1 TES delays photoreceptor and retinal ganglion cell loss

Retinitis pigmentosa (RP) is a genetic disorder characterized by the progressive degeneration of photoreceptors. There is currently no satisfactory cure for RP. It was reported that TES was able to delay photoreceptor loss and exerted a neuroprotective effect *via* modulating the electrical charge balance of the photoreceptors in the outer retina (Morimoto et al., 2007, 2012; Rahmani et al., 2013). Specifically, TES was shown to facilitate the survival of photoreceptors and preserved retinal function in the RCS (Royal College of Surgeons) rat model of RP (Morimoto et al., 2007). Furthermore, examination of the fundus at the end of the treatment showed there was no vitreous hemorrhaging or retinal detachment, indicating that TES had no side effects in the vitreous and retinal neurons of the RCS animal model. Another study also reported that TES had neuroprotective effects on the outer retinal neurons in a transgenic RP rabbit model (rhodopsin P347L; Morimoto et al., 2012). Interestingly, the results from the electroretinogram (ERG) experiments showed that TES preserved more cone cells than rod cells in the treated rabbits. Besides the therapeutic effects of TES on photoreceptors in the outer retina, several studies also reported TES had preservation effects in the inner retina. In another study in rats with optic nerve injury, TES at optimal parameters (current amplitude: 100–200 μA; stimulation frequency: 1, 5, or 20 Hz; pulse duration: 1–2 ms/phase and stimulation duration: above 30 min) was able to protect retinal ganglion cells (RGCs) and their axons (Tagami et al., 2009; Morimoto et al., 2010). The neuroprotective effects of TES on RGCs have also been demonstrated in rodent models of glaucoma (Jassim et al., 2021), ischemic retinal disease (Wang et al., 2011), optic nerve crush (Miyake et al., 2007; Henrich-Noack et al., 2013a, b), and light-induced retinal injury.

### 2.2. TES inhibits apoptosis and promotes neuronal regeneration

Studies on TES in healthy retina have also been performed to decipher the underlying mechanism of the therapeutic effects of TES on retinal degeneration. One study analyzed changes in the transcriptome of healthy rat retinas at 4 h after the application of TES (current amplitude: 200 μA, stimulation frequency: 20 Hz, pulse width: 1 ms/phase, duration: 60 min), which identified a total of 404 downregulated genes and 286 upregulated genes related to cell growth, cell signaling, tissue development, and proliferation (Willmann et al., 2011). Another study observed significant downregulation of pro-apoptotic protein factors such as B-cell lymphoma protein 2-associated X (Bax) and tumor necrosis factor (Kulsoom et al., 2018). These results suggest that TES inhibits cellular apoptosis. Furthermore, TES was reported to activate neuronal regeneration in the retina *via* promoting the secretion of neurotrophic factors, which are a class of protein molecules that facilitate neuronal growth, development, survival, and regeneration (Hempstead, 2006; Reichardt, 2006). In animal models of retinal photoreceptor degeneration (e.g., N-methyl-N-nitrosourea-treated mice and bright blue light-exposed rats), TES was shown to trigger the upregulation of brain-derived neurotrophic factor and ciliary neurotrophic factor (Ni et al., 2009; Tao et al., 2016).

### 2.3. TES promotes functional improvements in human retina

The beneficial effects of TES in the retina have also been observed in human studies. In a prospective randomized clinical trial involving 24 RP subjects, the weekly application of TES for six consecutive sessions resulted in positive effects on the visual field area and scotopic ERG compared to sham matched controls (Schatz et al., 2011). A follow-up study of 52 weeks of consecutive TES treatment in 52 RP patients also reported significant improvement in the photopic ERG responses of cone cells. A similar clinical study involving the application of TES in patients with retinal artery occlusion showed photoreceptor function was improved, as demonstrated by an increase in scotopic a-wave slope (Naycheva et al., 2013). Several other clinical studies also reported TES improved visual acuity, multifocal ERG, and visual field area in patients with retinal artery occlusion (Inomata et al., 2007; Oono et al., 2011), non-arteritic ischemic optic neuropathy, and traumatic optic neuropathy (Fujikado et al., 2006). In all the aforementioned clinical studies, TES was found to be well tolerated with no adverse side effects, supporting its safety as a potential therapeutic modality.

Recent advancements have extended the application of TES from restoring vision in the retina to activating brain neuronal networks. The modulation of the brain by TES is possible because of the unique eye-brain connection, as discussed below.

## 3 The eye-brain connection

**Figure 2:**
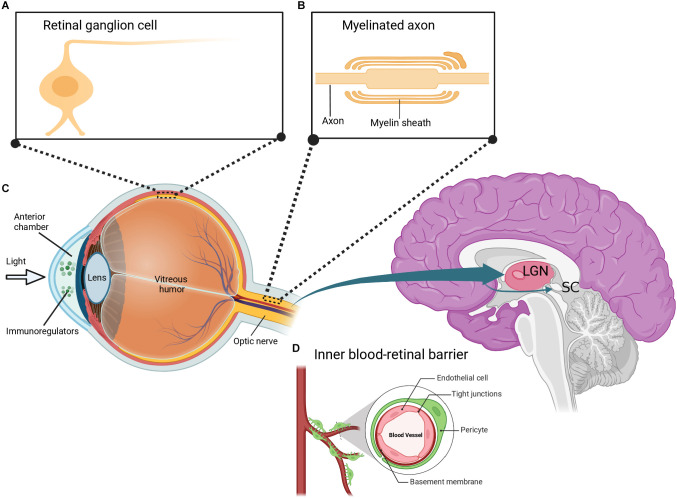
Depiction of the eye-brain connection. **(A)** The retina contains several layers of neurons including retinal ganglion cells (RGCs), which are morphologically similar to neurons in the brain. **(B)** RGCs have myelinated axons that are organized to form the optic nerve. The optic nerve extends to the lateral geniculate nucleus (LGN) and superior colliculus (SC) in the brain. **(C)** Similar to the brain, the eye is uniquely associated with the immune system and is composed of immunoregulatory molecules. **(D)** The inner blood-retinal barrier, which is analogous to the blood-brain barrier, further shows the relationship between the eye and the immune system. Created with BioRender.com (accessed December 13, 2022).

Over the years, scientists have been looking for scientific evidence on the usefulness of eye research in studying the brain and its diseases. Many studies have begun exploring this eye-brain connection in terms of clinical diagnosis and therapeutic interventions for brain diseases. The concept that “our eyes are the windows to our souls” is intriguing in that our eyes provide an insight into our brain health both neurologically and optically. It is well known that the retina and the optic nerve are part of the CNS, as both structures extend from the diencephalon during embryonic development. The retina is a transparent tissue composed of specialized neurons such as RGCs arranged in layers interconnected by synapses (Figure 2A). Axons of RGCs form the optic nerve and extend to both the lateral geniculate nucleus (LGN) of the thalamus and the superior colliculus (SC) of the midbrain (Figure 2B; Dhande and Huberman, 2014). Visible light entering the eye is captured by light-sensitive photoreceptor cells in the outer retina. This process triggers a signal cascade that transduces visual information into electrical signals that move along the inner retina to the RGCs. These electrical signals are then relayed to higher visual centers responsible for proper interpretation and visual perception. It is crucial to state that although majority of the RGCs form outputs that project to the LGN and SC, there is a subgroup of specialized RGCs that also project into the suprachiasmatic nucleus of the hypothalamus. It has been noted that this region plays a key role in the sleep/wake cycle and circadian rhythms (Fernandez et al., 2016). Furthermore, other RGCs project their axons to the pretectum of the midbrain, which communicates with the motor nuclei to exert pupillary control (Levine and Schwartz, 2020).

Despite differences in the morphology of RGCs and CNS neurons, RGCs retain the typical properties of brain neurons including characteristic neuronal cell body, dendrites, and axons (Berson, 2008). The optic nerve formed by the axons of RGCs also retains fiber tracts similar to that of brain neurons. Specifically, the optic nerve is covered with a myelin sheath and enclosed in three protective meninges (Figure 2B). Similar to brain axons, trauma of the optic nerve leads to retrograde and anterograde degeneration of the injured axons. Consequently, this triggers other destructive processes including oxidative stress, myelin destruction, scar formation, inhibition of neurotrophic factors, accumulation of protein aggregates, and secondary degeneration such as the death of neighboring neurons (Levkovitch-Verbin et al., 2001, 2003; Herro and Lam, 2015; Yu-Wai-Man, 2015). It has been reported that the regeneration of damaged axons is restricted in both the brain and the optic nerve. It has also been demonstrated that the neurons in the brain and the optic nerve share similar factors that result in an internal environment that limits axonal growth following injury. Some of these factors include myelin-associated inhibitors (Nogo A), myelin debris, reactive astrocytes, and chondroitin sulphate proteoglycans (Maxwell, 2013; Pernet, 2017; Guttenplan et al., 2020; Pearson et al., 2020; Yu et al., 2021a; Fawcett and Kwok, 2022; Hammel et al., 2022). Interestingly, a significant amount of information about the axonal response to brain injury was initially discovered in studies on the optic nerve (Benowitz and Yin, 2008; Fischer, 2012; Fischer and Leibinger, 2012; Heskamp et al., 2013; Benowitz et al., 2017; Fischer et al., 2017). More recent studies have reported that signaling in complex circuitry involving the blockage of inhibitory neurotransmission of retinal amacrine cells plays a crucial role in RGC axonal regeneration after optic nerve injury (Yin et al., 2019; Sergeeva et al., 2021).

As an integral part of the CNS, the eye also interacts with the immune system. The eyes contain several immunoregulatory molecules including cytokines and other surface molecules (Figure 2C) that induce specialized immune responses similar to those of neurons in the brain and spinal cord (Streilein, 2003; Kaur et al., 2008). The anterior part of the eye contains a fluid called the aqueous humor, which is enriched in anti-inflammatory and immunoregulatory factors and is similar to the cerebrospinal fluid (CSF) that bathes the brain and spinal cord (Kaur et al., 2008; Aleci et al., 2015). Indeed, the eye is surrounded by two main blood-ocular barriers, the blood-aqueous barrier, and the blood-retinal barrier, which share similar structural characteristics and mechanisms to the blood-brain barrier. The inner regions of the blood-retinal barrier are composed of specialized endothelial cells linked by tight junctions surrounded by pericytes (Figure 2D), astrocytes, and Müller cell end-feet, which closely resembles the blood-brain barrier (Kaur et al., 2008).

As the eyes are a prominent extension of the brain, it is gradually becoming common practice to examine ocular manifestations when diagnosing brain pathologies. The eye-brain connection suggests the investigation of the eye could have clinical value in the early diagnosis of brain pathologies. Interestingly, results from ophthalmological assessments have revealed that several neurodegenerative disorders and neurological diseases such as AD, PD, and multiple sclerosis have manifestations in the retina. Similar to the pathological features in the brain of patients with AD, Aβ and p-tau can be present in the eye and are associated with ocular damage including cataract formation, retinal nerve fiber layer (RNFL) thinning, and retinal neuronal death (Goldstein et al., 2003; Ning et al., 2008; Liu et al., 2009; Gasparini et al., 2011; Chiu et al., 2012). In patients with PD, photoreceptors, and RGCs have been observed to display distinct swelling accompanied with morphological thinning of the RNFL (Moschos et al., 2011; Kirbas et al., 2013; Chang et al., 2022). In up to 50% of patients with multiple sclerosis, loss of vision has been reported as a presenting symptom, although other cases can also involve some degree of visual impairment. It is important to note that the aforementioned eye manifestations may not be specifically related to a particular disease, although their presence is a strong argument for the connection between the retina and the brain. More importantly, the direct connection to the brain *via* the long continuous tract of the optic nerve makes the eye ideally suited for stimulation by non-invasive approaches to exert neuromodulatory effects in the brain.

## 4 Neuromodulatory effect of TES on the brain

### 4.1. Evidence from electrophysiological and behavioral studies

There are many studies on the use of TES in ophthalmology (Morimoto et al., 2005; Ni et al., 2009; Tagami et al., 2009; Wang et al., 2011; Naycheva et al., 2013; Fu et al., 2018; Jassim et al., 2021), but relatively fewer studies have explored its effects on the brain. It is gradually becoming clearer that TES not only affects the retina and its related structures, but can also modulate neurons in the brain. Interestingly, TES has been demonstrated to stimulate not only vision-related brain regions, but also other regions not directly related to visual processing. In a human study, ^18^F-fluorodeoxyglucose positron emission tomography clearly showed that TES activated vision-related brain regions, including the occipital cortex (primary and secondary visual cortex respectively) and inferior temporal gyrus (Xie et al., 2011). Furthermore, TES also enhanced neuronal network activity in non-visual streams, such as the bilateral prefrontal cortex (PFC) and parahippocampal gyrus. In animals, the neuromodulatory effects of TES have also been investigated in healthy and visually impaired rodents using EEG and ECoG recording techniques. In healthy rats, TES was reported to cause EEG after-effects in the primary visual cortex (V1). Significantly increased theta oscillation power was observed in V1 area that lasted 15 min beyond the TES treatment period (Sergeeva et al., 2012). The effects of prolonged TES have also been reported in retinal degeneration (rd) mice models under varying stimulation parameters. Similar results were observed in the brains of both blind rd mice models and healthy rodents. Separate studies using different parameters including stimulation current, stimulation frequency, pulse duration, and repeat application (30 min/day for 1 week) reported TES significantly increased the power, coherence, brain connectivity, and oscillatory cross-frequency coupling of ECoG electrophysiological recordings of V1 and PFC in the brains of rd10 mice compared to sham matched controls. Specifically, the oscillations modulated by TES were predominantly in the low frequency range of the ECoG bands such as delta (1–5 Hz), theta (5–10 Hz), alpha (10–15 Hz), and beta (15–30 Hz) oscillations (Agadagba et al., 2019, 2020, 2021, 2022; Agadagba and Chan, 2020, 2021). A peculiar observation is that TES appeared to activate the brain region of interest in both healthy and blind rodents, with the resulting neuromodulation maintained beyond the stimulation period. For healthy rodents, the maintenance period was 15 min after the TES, whereas for rd rodents, the effect persisted for a few weeks. Low frequency oscillations (LFOs) have been suggested to facilitate neural processing *via* modulating neuronal excitability periodicity (Herrmann and Henry, 2014; Hickey and Race, 2021). Furthermore, LFOs have been suggested to be important indicators of neuroplasticity (Assenza and Di Lazzaro, 2015; Cassidy et al., 2020). In regards to the association between LFOs, neuronal processing, and neuroplasticity, the results from the aforementioned studies support that TES has neuroplasticity effects in healthy and diseased animals, respectively. Moreover, improved neuroplasticity was reported to be a common mechanism of action of various brain stimulation methods (Kandel, 2001; Hogan et al., 2020). Therefore, in addition to its ability to alter brain neuronal network activity, TES may also facilitate long-term potentiation. The neuroplasticity potential of TES in rodent animals was confirmed in an optic nerve lesion study in humans, which reported a significant post-TES increase in EEG alpha oscillations in the occipital visual cortex (Sabel et al., 2011). Although the authors did not explicitly provide an actual mechanism for the observed clinical effects of the stimulation, they proposed that the induction of synaptic plasticity and neuronal network synchronization could be responsible for mediating the EEG changes. Furthermore, a study using tetrodotoxin (a sodium ion channel blocker) to block RGC activity, which also consequently blocked V1 electrical evoked potentials, showed that TES could stimulate RGCs to activate the occipital visual cortex *via* the generation and transmission of action potentials in the retinogeniculate pathway (Foik et al., 2015). The authors suggested that during TES, action potential generation in normal RGCs activated evoked potentials in V1. Moreover, the finding further strengthens the idea that TES can induce the neuromodulation of brain activity *via* the promotion of excitatory action potentials as well as synchronize neuronal activity. It is possible that repetitive application of TES could repeatedly activate the brain to strengthen synapses and facilitate neuronal information processing/transfer, ultimately leading to functional improvements in visual and non-visual brain regions. Besides the aforementioned pathways, other processes such as changes in the regulation of relevant neurotransmitters may also contribute to the beneficial effects of TES on brain networks. However, such mechanisms remain to be explored.

The functional effects of TES in modulating neurons in the brain have also been investigated in several rodent behavioral studies. These studies reported that TES changed the behavioral patterns of experimental animals, which were positively correlated with the activation of certain brain regions. For example, three pioneering studies on TES demonstrated altered behaviors in corneally kindled rats (Wlaź et al., 2015; Koshal and Kumar, 2016; Albertini et al., 2018). Kindling is the progressive increase in electrographic and behavioral seizure activity induced by the initial application of a sub-convulsive electrical current to limbic brain regions such as the hippocampus and amygdala. In the aforementioned studies, corneal kindling was performed by repeated sub-convulsive doses of TES until a generalized seizure was attained, which induced epilepsy-like behaviors in rodent animals. In the forced swim immobility behavioral test, fully kindled rats exhibited a significant reduction in despair-related behavior following TES (Wlaź et al., 2015). However, in the elevated plus maze test, there was a marked increase in anxiety-like behavior following TES. Interestingly, the anxiety-like behavior was not observed in rats with 6 Hz stimulation, rather an anhedonic response was recorded in the novelty suppressed feeding and saccharin preference behavioral tests (Albertini et al., 2018). Nevertheless, these studies show that TES can modulate the brain network as demonstrated by the altered behaviors of rodents. Conversely, it should be noted that a downside of corneal kindling is that the application of high-intensity electrical stimulation (up to 19 mA amplitude) can result in high mortality rates in the experimental animals (Yu et al., 2021b). Thus, the kindling protocol cannot be readily translated to human clinical trials.

More recently, some behavioral studies in rodent models have extended the exploration of the effects of TES on brain neurons to the therapeutic potential of TES on depression. A study examined the potential antidepressant-like effects of TES (Yu et al., 2022a) administered daily for 1 week in S334ter-line-3 rats and chronic unpredictable stress (CUS) rats. Notably, S334ter-line-3 rats, which are commonly used in the rd animal model, were used for the animal model of depression in this study, considering that vision loss can make blind patients more vulnerable to develop anxiety and depression (Hahm et al., 2008; Zheng et al., 2017; Frank et al., 2019). The S334ter-line-3 rats exhibited heightened levels of anxiety in the behavioral testing. Following TES, the S334ter-line-3 rat group displayed anxiolytic and antidepressant-like behaviors in the forced swim test, cylinder test, open field test, and home cage emergence test. In the CUS rat group, TES significantly decreased behavioral despair and increased hedonic-like behaviors. Treatment with temozolomide (a neurogenesis blocker) only blocked the hedonic-like behavioral effect of TES in the experimental rats, which suggests the antidepressant-like effects of TES were partly mediated by a neurogenesis-dependent pathway.

### 4.2. Evidence from molecular studies

Several molecular studies have investigated the neuroprotective effect of TES mainly in retinal neurons. Some studies have also begun to explore the neuromodulatory effects of TES on brain neurons at the molecular level. Given that chronic stress-induced disruption of neuroplasticity has been implicated in the development of depression (Pittenger and Duman, 2008), some molecular studies have focused on the neurogenic potential of TES on neurogenesis, synaptic plasticity, and apoptosis in rodent models of depression and anxiety. The hippocampus of rats treated with TES showed upregulated protein biomarkers of progenitor cell proliferation including Ki67 and Nestin (Sun et al., 2014; Yu et al., 2022a), but other biomarkers of neurogenesis such as Doublecortin (Dcx) and NeuN were not significantly changed. A possible reason for the latter result proposed by the authors was that hippocampal progenitor cells in adult rat usually differentiate and mature by 3–5 weeks (Kohler et al., 2011), but the animals in this study were sacrificed at the early stage of proliferation, hence, the cells expressed lower levels of Dcx and NeuN.

In depression, there is significantly altered apoptosis and synaptic plasticity in the limbic system especially in the hippocampus and amygdala (Pittenger and Duman, 2008). Functional connections between the limbic system and the cortical regions of the brain, including the visual cortex, PFC and inferior temporal cortex, suggest the involvement of the hippocampus and amygdala in processing visual-emotional stimuli. Synaptophysin (SYP), a synaptic vesicle protein, has been reported to regulate synaptic plasticity *via* activity-dependent synapse formation (Tarsa and Goda, 2002). Moreover, postsynaptic density (PSD)-95 has also been implicated in synaptic plasticity of glutamatergic synapses during neurodevelopment (Coley and Gao, 2018). Several studies using the CUS rat model of depression reported abnormal expression levels of presynaptic SYP in the hippocampus and amygdala (Zhang et al., 2014; Kang et al., 2020; Ma et al., 2021; Woodburn et al., 2021), and dysfunctional expression of postsynaptic biomarker PSD95 in the amygdala (Zhang et al., 2018; Yu et al., 2022b). Treatment with TES significantly normalized the dysfunctional expression of SYP and PSD95 in the hippocampus and amygdala of CUS rats (Yu et al., 2022a). Interestingly, these results suggest that TES can modulate synaptic function to induce antidepressant-like responses in the CUS rat model of depression.

As mentioned earlier, TES can decrease the expression of the pro-apoptotic protein Bax in the retina (Ni et al., 2009; Willmann et al., 2011; Kulsoom et al., 2018). Similar anti-apoptotic effects were also reported in the brains of CUS rats after the application of TES (Yu et al., 2022a) or the administration of antidepressants (Ding et al., 2014; Colla et al., 2021). Protein kinase B or AKT are widely expressed in the limbic circuit and play diverse roles in glucose metabolism, cell proliferation, and neuronal apoptosis. Notably, phosphorylation of AKT at its Ser-473 inhibits cellular apoptosis by preventing the release of cytochrome c from mitochondria (Zhou et al., 2000). However, studies involving animal models of stress showed contrasting results for AKT in the hippocampus and amygdala. Some studies reported decreased AKT expression arising from stress and depression (Leibrock et al., 2013; Wu et al., 2021), whereas other studies reported increased AKT expression levels (Lee et al., 2006; Yang et al., 2008; Eagle et al., 2013). In CUS rats, TES was shown to modulate the AKT circuitry by normalizing the expression of phosphorylated AKT in the hippocampus. Protein kinase A (PKA) is a major regulator of several metabolic processes involving cyclic adenosine monophosphate. Given its crucial role in cellular metabolism, PKA has also been implicated in neuronal survival (Dwivedi and Pandey, 2008). Reduced levels of neuronal PKA were observed in both post-mortem analysis of brains from depressed patients (Shelton et al., 2009) and in social defeat stress models of depression (Chen et al., 2012). The anxiety-like behaviors were reported to be alleviated following the administration of PKA activators in the amygdala (Yang et al., 2016). Interestingly, TES was also able to restore the reduced PKA activity in the amygdala of CUS rats (Yu et al., 2022a).

## 5 Implications of TES as a non-invasive neuromodulatory tool for modulating brain activity

There are numerous unexplored neuronal pathways in the brain which could serve as potential targets for disease treatments and for enhancing brain function. Non-invasive neuromodulation techniques such as TES can alter brain activity and are rapidly becoming popular research tools due to their safety and not requiring surgery. Besides activating retinal neurons, TES can also modulate neuronal excitability in the brain. Preclinical studies have shown that TES can attenuate depression and anxiety-related symptoms in rodent models (Yu et al., 2019, 2021b, 2022a). Moreover, TES is adjustable depending on the disease type and disease progression. Standard anti-depressants affect entire biological systems and can lead to adverse side effects such as memory impairment, decreased concentration, and weight gain. Conversely, TES is generally safe without any serious side effects. Both short-term and prolonged TES in human subjects is generally well tolerated, although some minor and transitory side effects such as dry eyes, foreign body sensation, and corneal punctate keratopathy have been reported (Fujikado et al., 2006; Morimoto et al., 2006; Wagner et al., 2017; Jolly et al., 2020). Cases of adverse effects after TES have been rare and were possibly due to the high amplitude stimulation or mechanical pressure (Ni et al., 2009). Nonetheless, because of the simplicity of TES administration, the stimulator can be promptly turned off and the stimulating electrode removed in the rare case of adverse effects.

Besides its potential for treating depression, TES has also been explored as a potential therapy for cognitive dysfunction. In a recent study in aged mice and 5XFAD mice (AD model) treated with 3 weeks of TES, there were improvements in hippocampal-dependent learning and memory performance in both groups of mice (Yu et al., 2022b). Subsequent hippocampal immunostaining and protein expression analysis in 5XFAD mice showed that male mice had significantly reduced hippocampal plaque deposition and upregulated levels of PSD-95 protein. Although this suggests that the neuromodulatory effects of TES involve a postsynaptic mechanism, it is also interesting to note that elevated levels of PSD-95 are reported to protect synapses from amyloid-beta plaques (Shao et al., 2011; Yuki et al., 2014; Dore et al., 2021). Future studies on TES in other preclinical models of dementia are needed to investigate its beneficial effects on cognitive deficits and to delineate the underlying mechanisms.

Another future application is the use of TES as a non-invasive neuromodulatory tool for activating the brain to enhance vision-related behaviors, specifically visual attention. Previous studies involving the application of non-invasive tDCs in healthy subjects reported the modulation of spatial attention *via* altering the neuronal network activity ratios between the two brain hemispheres (Ni et al., 2019; Andres et al., 2020). Furthermore, electrophysiological studies reported that tDCs enhanced cognitive control and improved visual attention tasks following entrainment with alpha oscillations (Clayton et al., 2019). Similarly, prolonged TES in rd10 mice resulted in significantly increased ECoG alpha oscillations in V1 (Agadagba et al., 2021, 2022). Increased spontaneous firing rate in V1 neurons is known to be a pathological feature of rd. Alpha oscillations have also been noted to have an inhibitory role that helps the brain to focus on and process more relevant neural signals while ignoring less relevant signals. The above studies on tDCs parallel the rd10 study, which implies TES could be a beneficial tool for vision-related studies, especially studies focused on the functional rehabilitation of patients suffering from visual neglect.

This review highlights the neuromodulatory effects of TES in the brain *via* the unique eye-brain architecture. It is clear that TES has rapidly advanced from regulating retinal neurons to a cutting-edge research tool for modulating both visual and non-visual brain regions with beneficial outcomes in both healthy and diseased subjects. There is also ample experimental evidence from electrophysiological, behavioral, and molecular studies to support the potential use of TES as a non-invasive neuromodulatory tool for enhancing brain activity. Possible future directions of TES research include the mechanistic study of its neuromodulatory effects and further examining the cognitive and perceptual processes involved. Such mechanistic studies need to be properly designed with specific controls to reduce the generalizability of findings and to optimize the TES stimulation parameters for translation to human clinical trials.

## Author contributions

All authors (SA, LL, and LC) contributed to the article and approved the submitted version.
